# Seeding and feeding milestones: the role of human milk microbes and oligosaccharides in the temporal development of infant gut microbiota

**DOI:** 10.1017/gmb.2024.5

**Published:** 2024-05-31

**Authors:** Martha F. Endika, David J. M. Barnett, Cynthia E. Klostermann, Noortje Kok, Henk A. Schols, Arjen Nauta, Ilja C. W. Arts, John Penders, Koen Venema, Hauke Smidt

**Affiliations:** 1 Laboratory of Microbiology, Wageningen University & Research, Wageningen, The Netherlands; 2 Maastricht Centre for Systems Biology (MaCSBio), Maastricht University, Maastricht, The Netherlands; 3 Department of Medical Microbiology, Infectious Diseases and Infection Prevention, Maastricht University Medical Center+, Maastricht, The Netherlands; 4 Biobased Chemistry and Technology, Wageningen University & Research, Wageningen, The Netherlands; 5 Laboratory of Food Chemistry, Wageningen University & Research, Wageningen, The Netherlands; 6 FrieslandCampina, Amersfoort, The Netherlands; 7 Centre for Healthy Eating & Food Innovation (HEFI), Maastricht University – campus Venlo, Venlo, The Netherlands

**Keywords:** breastfeeding, HMO, bacterial composition, faeces, mother–infant pairs

## Abstract

Breastfeeding represents a strong selective factor for shaping the infant gut microbiota. Besides providing nutritional requirements for the infant, human milk is a key source of oligosaccharides, human milk oligosaccharides (HMOs), and diverse microbes in early life. This study aimed to evaluate the influence of human milk microbiota and oligosaccharides on the composition of infant faecal microbiota at one, three, and nine months postpartum. We profiled milk microbiota, HMOs, and infant faecal microbiota from 23 mother–infant pairs at these time points. The predominant genera in milk samples were *Streptococcus*, *Staphylococcus*, and an unclassified *Enterobacteriaceae* genus-level taxon (*Enterobacteriaceae* uncl.), whereas the infant faecal microbiota was predominated by *Bifidobacterium, Bacteroides*, and *Enterobacteriaceae* uncl. Mother–infant dyads frequently shared bacterial amplicon sequence variants (ASVs) belonging to the genera *Bifidobacterium, Streptococcus, Enterobacteriaceae* uncl.*, Veillonella, Bacteroides*, and *Haemophilus.* The individual HMO concentrations in the milk showed either no change or decreased over the lactation period, except for 3-fucosyllactose (3-FL), which increased. Neither maternal secretor status nor HMO concentrations were significantly associated with microbiota composition at the different ages or the bacterial ASVs of maternal milk and infant faeces. This study suggests an age-dependent role of milk microbes in shaping the gut microbiota, while variations in HMO concentrations show limited influence.

## Introduction

Breastfeeding is one of the most important drivers of gut microbiota development in early life (Bäckhed et al., 2015; Azad et al., 2016; Stewart et al., 2018). Human milk contains rich nutrient resources and is a source of diverse microbes, containing between 10^2^ and 10^5^ viable bacteria per ml (Martín et al., 2003; Jost et al., 2013; Schwab et al., 2019). Human milk also contains a high concentration of structurally diverse non-digestible oligosaccharides, human milk oligosaccharides (HMOs), in the range of 4–22 g/L that vary geographically between individuals and over lactation stages, as previously reviewed (McGuire et al., 2017; Thum et al., 2021). Moreover, variations in the HMO profile are dependent on the expression of the maternal secretor (Se) and Lewis (Le) genes, which determine the maternal secretor status and Lewis blood group, as well as structural composition of fucosylated HMOs (Thurl et al., 1997). The milk of secretor (Se+) mothers contains an abundance of α1,2-fucosylated HMOs, such as 2′-fucosylactose (2′-FL), while the milk of non-secretor (Se-) mothers lacks this HMO group due to the loss of fucosyltransferase 2 enzyme activity (Cabrera-Rubio et al., 2019). Additionally, the genetic variations in *FUT3*, which define Lewis status, were correlated with the concentration of α1,4-fucosylated HMOs, for example lacto-N-fucopentaose II (LNFP II) (Lefebvre et al., 2020). The majority of ingested HMOs reaches the colon and provides selective substrates for the growth of HMO-utilizing bacteria, including members of *Bifidobacterium* and *Bacteroides* that are both predominant genera in the gut microbiota of breastfed infants (Marcobal et al., 2010; Yu et al., 2013).

In the first year of life, the compositional changes in human milk microbiota and HMOs occur alongside the temporal development of the infant gut microbiota (Liu et al., 2022). The associations between HMO concentrations and the faecal microbiota of breastfed Dutch infants during the first 12 weeks of life were previously investigated (Borewicz et al., 2020). However, only a few studies focused on the role of breastfeeding, particularly as source of both milk microbes and oligosaccharides, in the development of gut microbiota across the lactation period. Most studies have investigated this based on observations with two time points from the group of infants older than 3 months of age in Canadian and Danish populations (Fehr et al., 2020; Laursen et al., 2021), while none have been reported from the Dutch population.

A previous study observed geographic variations in HMOs among ethnically similar mothers, suggesting environmental and dietary influences (Thum et al., 2021). Additionally, dietary habits are strongly influenced by cultural factors and country of birth, and maternal diet holds the potential to impact the microbiota in human milk and the infant gut, as previously reviewed (Taylor et al., 2023). Moreover, cultural differences related to solid food introduction were identified between Dutch and Canadian populations (Homann et al., 2021). In the Netherlands, children are introduced to complementary food from the age of four months and their growth and development are monitored through eight visits to local child health clinics during the first year, including appointments at one, three, and nine months after birth. Therefore, we aimed to evaluate the influence of microbes and HMO concentrations in the breast milk of Dutch mothers on the development of infant gut microbiota throughout the lactation period at one, three, and nine months of age.

## Methods

### Study design and sample collection

**Figure 1.**
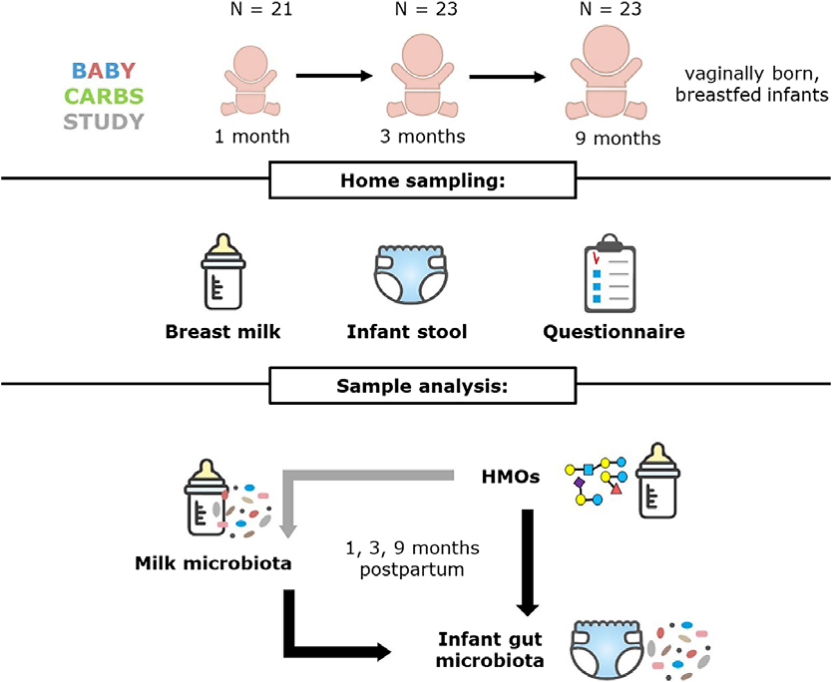
Overview of Baby Carbs study design and sample collection.

The current study included breast milk and infant faecal samples collected from mother–infant pairs who participated in the Baby Carbs study (Figure 1). Healthy, vaginally delivered, full-term Caucasian infants whose mothers intended to exclusively breastfeed at least up to three months after birth were eligible for the study. Exclusion criteria included preterm birth (< 37 weeks of gestation) and infants who received antibiotics during their first month of life. This study was exempted from medical research ethics committee approval for the collection of samples, after review by the Medical Ethical Reviewing Committee of Wageningen University. All parents provided written informed consent before the start of the sample collection. Sample collection was scheduled at one, three, and nine months postpartum.

The participants collected up to 20 ml of breast milk in the morning before feeding their child (foremilk), without cleaning the breast. The milk samples were collected without aseptic cleansing in order to provide a more representative analysis of the microbiota, present in breast milk and on the breast surface, as ingested by the suckling infant during breastfeeding (Sakwinska et al., 2016; Simpson et al., 2018). The milk was collected by hand expression or using a breast pump into a sterile 50-ml tube (Greiner Bio-One™ CellStar™ test tubes, Alphen aan den Rijn, the Netherlands) and kept in the home refrigerator for a maximum of 5 h prior to the visit from one of the researchers. Infant faecal samples were collected from a diaper using a sterile spoon (Sampling Systems, Coleshill, United Kingdom). The collected faeces were kept anoxically in a sterile 50-ml collection tube with filter cap (Greiner Bio-One™ Cellreactor™ tubes, Alphen aan den Rijn, the Netherlands) placed inside BD GasPak EZ anaerobe gas-generating pouches (BD Diagnostics, Sparks, MD, United States). These faecal samples were stored in the home refrigerator at approximately 4 °C for a maximum of 72 h before being collected by one of the researchers for transport to the laboratory. The samples, both breast milk and infant faeces, were transported to the laboratory inside an insulated bag containing frozen cooling elements and stored at −80 °C until further processing.

### DNA extraction from breast milk samples

The milk samples were thawed at room temperature and subsequently centrifuged (10,000× g, 10 min, 4 °C). The aqueous fraction was then transferred to a new Eppendorf tube for further analysis of HMO structures. The DNA extraction from breast milk was performed based on the method previously described by Schwab et al. (2019). Briefly, the cell pellet including the fat layer was used for DNA extraction to identify the overall prokaryotic profile in breast milk (Stinson et al., 2021). The milk DNA was extracted using the FastDNA spin kit for soil (MP Biomedical, Eschwege, Germany) following the manufacturer’s protocol, which includes a bead-beating step. Each DNA extraction batch included milk samples and negative control (buffer only).

### HMO analysis

HMOs were isolated from milk and purified based on a method slightly modified from the protocol described by Gu et al. (2021), using Supelclean ENVI-Carb 250-mg/3-ml solid-phase extraction (SPE) cartridges (Merck, Darmstadt, Germany). Two fractions were extracted, the first fraction (fraction A) containing 3-fucosyllactose (3-FL) as eluted by 3% acetonitrile (ACN) and the second fraction containing other HMOs (fraction B) eluted using 40% acetonitrile with 0.05% trifluoroacetic acid. Both fractions were subsequently evaporated to dryness using Eppendorf Concentrator Plus (Eppendorf Nederland BV, Nijmegen, the Netherlands) overnight at room temperature and then rehydrated in Milli-Q water for further analysis.

In total, 18 HMO structures were analysed (see Supplementary Table 1 for full HMO names), including eight fucosylated HMOs (3-FL, 2′-FL, LNFP I, LNFP II, LNFP III, LNFP V, LNDFH I, and DFL), four non-fucosylated neutral HMOs (LNT, LNnT, LNH, and LNnH) and six sialylated HMOs (3′-SL, 6′-SL, LST a, LST b, LST c, and DSLNT). The quantification of 3-FL (fraction A) and DSLNT (fraction B) was performed using high-performance anion-exchange chromatography-pulsed amperometric detection (HPAEC-PAD). For HPAEC, a gradient of two eluents was used, namely 0.1 M NaOH (eluent A) and 1 M NaOAc in 0.1 M NaOH (eluent B). The gradient for detection of 3-FL included 0–15% B (0–15 min), 15–100% B (15–20 min), and 100% B (20–25 min), followed by 20 min re-equilibration with 0% B. The gradient for detection of DSLNT included 0–25% B (0–25 min), 25–100% B (25–30 min), and 100% B (30–35 min), followed by 20 min re-equilibration with 0% B. Elution was performed at 0.3 ml/min at 25 °C. For quantification of other HMO structures, fraction B was further reduced to alditols using 0.5 M sodium borohydride, followed by SPE-based purification. The purified sample was analysed on a porous graphitized carbon-liquid chromatography mass spectrometry (PGC-LC-MS) equipped with a Thermo Hypercarb column (3 μm particle size, 2.1 mm × 150 mm; Hypercarb, Thermo Scientific, San Jose, CA, USA) in combination with a guard column (3 μm particle size, 2 mm × 10 mm; Hypercarb, Thermo Scientific).

The HMOs were identified by comparing the retention time and mass-to-charge ratios with commercial reference oligosaccharides (Supplementary Table 1). The total HMO concentration was calculated as the sum of the 18 identified HMOs. Maternal secretor status was classified based on the high concentration (secretor) or near absence (non-secretor) of 2′-FL with the lower quartile as a cut-off concentration (16.5 μg/ml). The Lewis status was classified based on the presence or absence of LNFP II (Wang et al., 2020).

### DNA extraction from infant faecal samples

Faecal DNA was extracted from 50–200 mg infant faeces that was re-suspended in 350 μl Stool Transport and Recovery (STAR) buffer (Roche Diagnostics, Indianapolis, IN, USA) and then transferred to a sterile screw cap tube (BIOplastics, Landgraaf, the Netherlands) containing 0.25 g of 0.1-mm zirconia beads and three glass beads (diameter 2.7 mm). The DNA extraction was performed following the repeated bead beating method (Salonen et al., 2010). Automated purification was performed using the Maxwell® 16 Tissue LEV Total RNA Purification Kit Cartridge customized for DNA purification (XAS1220) on the Maxwell® 16 Instrument (Promega, Madison, WI, USA).

### Microbiota analysis

The V4 region of the 16S rRNA gene was amplified in duplicate using barcoded 515F (Parada et al., 2016)- 806R (Apprill et al., 2015) primers. The full description of the PCR steps has been provided in a previous study (Endika et al., 2023). A total of 25 or 30 PCR cycles were used for faecal or milk samples, respectively. No-template controls were included for each PCR run. Duplicate PCR products were pooled for each sample and then purified by the use of the CleanPCR Kit (CleanNA, Waddinxveen, the Netherlands). Two mock communities of known 16S rRNA gene composition and one no-template control were included for each library. An equimolar mix of purified PCR products was prepared and sent for Illumina paired-end 150 bp Novaseq6000 sequencing at Novogene (Novogene-Europe, Cambridge, United Kingdom). The raw sequence data were processed using NG-Tax 2.0 with default settings (Poncheewin et al., 2020). Taxonomy was assigned based on SILVA database version 138.1 (Quast et al., 2012).

## Data analysis

Data analysis was performed in R version 4.2.0, and data were visualized using the microViz package version 0.10.8 (Barnett et al., 2021). Potential reagent contaminants in milk samples were identified based on either the frequency of amplicon sequence variants (ASVs) that varied inversely with sample DNA concentration or an increased prevalence of ASVs in negative controls using the decontam package version 1.17.0 (Davis et al., 2018). Subsequently, ASVs belonging to a list of known contaminant genera were removed (Salter et al., 2014). After processing, averages of 118,395 reads per milk sample and 271,775 reads per faecal sample were obtained. For alpha-diversity analyses, we used the exponent of Shannon index, calculated at genus level (effective Shannon Index). The Wilcoxon signed-rank test was performed to test differences in alpha-diversity between age groups using the rstatix package version 0.7.0 (Kassambara, 2021). Centred log-ratio (CLR)-transformed abundances at genus level were used in principal component analysis (PCA) scatterplots to visualize major patterns of microbiota variation. The binary Jaccard similarity index (presence–absence of shared ASVs), ranging from 0 (no shared ASVs) to 1 (all ASVs shared), was calculated to measure similarity between breast milk and infant faecal microbiota. For the statistical models, the HMO concentrations were transformed to z scores. PERMANOVA, using 9,999 permutations on the Aitchison distance, was performed to test the association of infant sex, birth place, milk collection method, and each HMO with age-specific microbiota at ASV level in milk or faeces. To explore associations between HMO exposures and the microbial relative abundances at ASV level, a simple linear regression model was used on the log2-transformed bacterial proportions (zeroes were replaced by half of the smallest observed value), per taxon, per age group. Only ASVs observed in more than five samples were included in the analysis. The p-values were corrected (Benjamini–Hochberg FDR-adjusted) per age group, for each HMO variable.

## Results

### Participant characteristics

In total, 23 infant–mother dyads participated in the study. Two infant–mother pairs could not provide samples at one month postpartum due to the restrictions related to the COVID-19 pandemic. Four mothers stopped breastfeeding their infants at nine months postpartum. All infants were born vaginally at term and exclusively breastfed at least for the first three months postpartum. Eleven infants were born at home, ten were born at a hospital, and two infants were born at a clinic. Of all infants, 52% were female and 56% were born after 40 weeks of gestational age (Supplementary Table 2). Regarding maternal secretor status, 17 mothers were classified as secretors and six mothers were classified as non-secretors. All milk samples contained LNFP II, indicating that all mothers in this study were Lewis-positive.

### Temporal dynamics in microbiota composition and taxa shared within the mother–infant pairs

**Figure 2.**
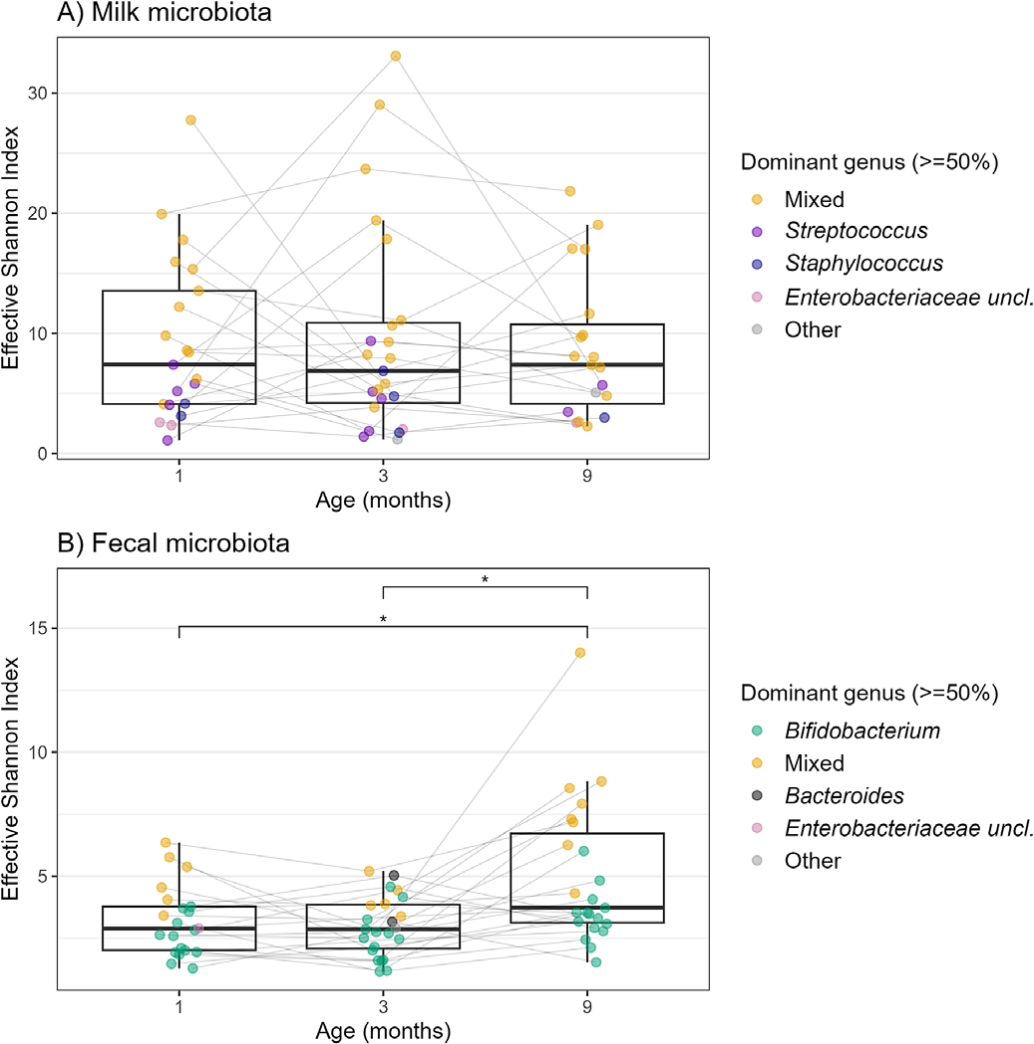
Alpha-diversity of microbiota in breast milk (A) and infant faeces (B) at different sampling moments. Boxplot (median and inter-quartile range) of alpha-diversity as measured by the effective Shannon index at genus level, grouped by age. Paired Wilcoxon signed-rank test was used to compare the diversity between two age groups. Significant differences are indicated by *p < 0.05.

We observed that breast milk samples were often dominated (defined as one genus accounting for at least 50% of reads from a given sample) by either *Streptococcus* or *Staphylococcus* or *Enterobacteriaceae* uncl.; however, the majority of samples were characterized by a mixed microbial composition (Figure 2A). On the other hand, most of the infant faecal samples showed a microbiota composition dominated by either *Bifidobacterium, Bacteroides*, or an unclassified genera within the *Enterobacteriaceae*, and relatively few faecal samples showed a mixed microbial composition (Figure 2B). The average alpha-diversity (effective Shannon index) of infant faecal samples at nine months postpartum was significantly higher than that of samples at one month (p = 0.011) and three months (p = 0.011) of age, while there was no significant difference in alpha-diversity in milk microbiota among different age groups (Figure 2).

**Figure 3.**
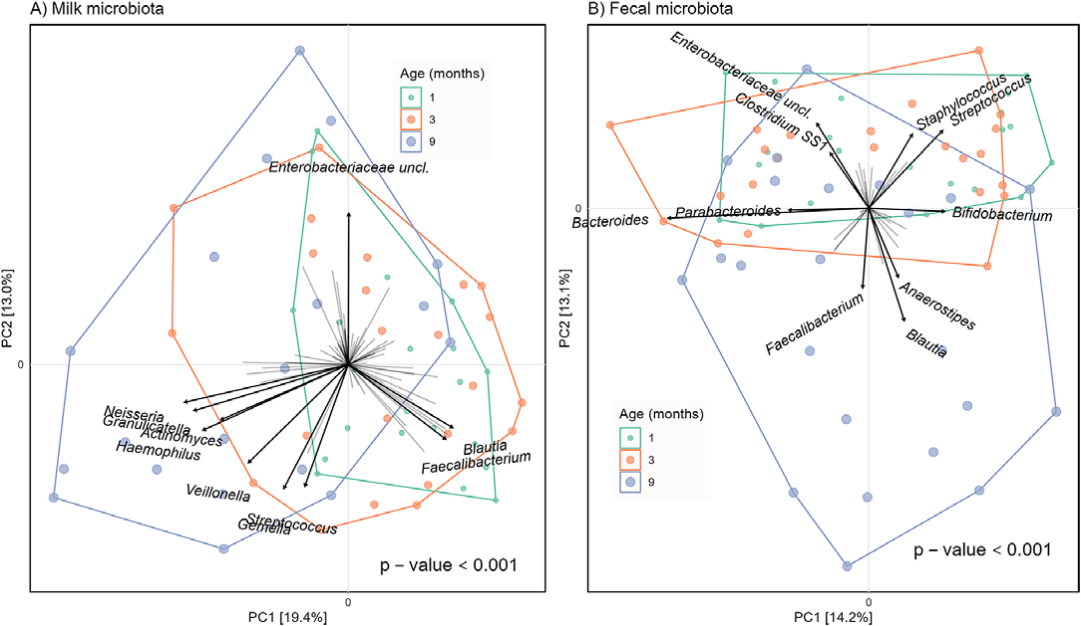
Beta-diversity of microbiota in breast milk and infant faeces at different sampling moments. PCA plots based on CLR-transformed microbial proportion at genus level. Taxon loading vectors are shown for 10 taxa that contributed most to the observed variation in microbial composition. Plots are coloured by age group, and the p-values shown are for the association of age with microbiota composition (PERMANOVA, Supplementary Table 3). Percentages at the PCA axes indicate the amount of variation explained. As a visual aid, convex hulls are drawn that connect the outermost data points for each age group.

We also observed temporal changes in the composition of both milk and faecal microbiota with a separation of some of the microbiota profiles at nine months of age from those observed for the younger age groups (Figure 3), especially for faecal microbiota profiles (Figure 3B). Age significantly explained 9% and 11% of the variance in milk and faecal microbiota, respectively, as determined by PERMANOVA (p < 0.001, Supplementary Table 3).

In the weaning period at nine months postpartum, the milk microbiota changed towards a community associated with increases in proportion of the bacterial genera *Neisseria, Granulicatella, Haemophilus, Actinomyces, Veillonella, Gemella*, and *Streptococcus* (Figure 3A). In the same period, the faecal microbiota changed towards a community associated with increases in the proportion of *Anaerostipes*, *Blautia*, and *Faecalibacterium* (Figure 3B). The changes over time in the proportion of taxa shown on the PCA plot are visualized in Supplementary Figure 1.

**Figure 4.**
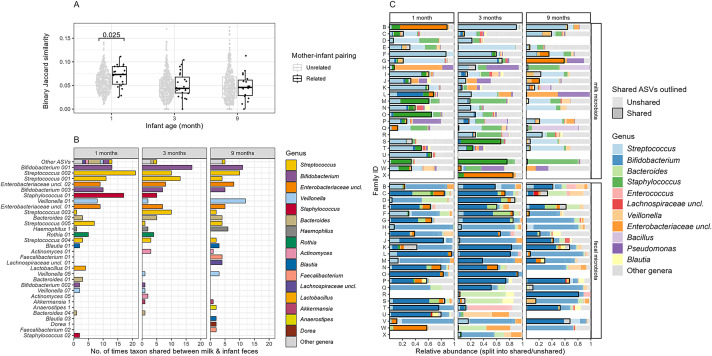
Shared ASVs between breast milk and infant faeces. (A) Boxplots of binary Jaccard similarity based on shared ASVs between milk and infant faeces at one, three, and nine months of age from related or unrelated mother–infant pairs. Significant differences are indicated by p-value <0.05. (B) Bar plot showing the number of times each bacterial ASV is shared between milk and infant faeces. (C) Bar plot showing the relative abundances of the bacterial ASVs that are shared or not shared within families (mother–infant pairs) for each sample (upper facets are milk sample compositions, lower facets are faecal sample compositions). ASVs belonging to the same genus are indicated by the same colour.

In order to evaluate the potential influence of bacteria from maternal breast milk in seeding the infant gut, we compared the shared bacterial ASVs between related and unrelated mother–infant pairs. This comparison revealed that the similarity between milk and infant faeces microbiota was higher within related mother–infant pairs than between mothers and unrelated infants at one month postpartum, whereas no difference was observed at three and nine months postpartum (Figure 4A). Furthermore, we observed that ASVs belonging to the genera *Bifidobacterium, Streptococcus, Enterobacteriaceae* uncl.*, Veillonella, Bacteroides*, and *Haemophilus* were frequently shared for all age groups (Figure 4B).

It should be noted that the high relative abundance of shared ASVs within the genus *Streptococcus* and *Staphylococcus* in milk did not correspond to a high relative abundance of these ASVs in infant faecal samples (Figure 4C). On the other hand, the shared ASVs belonging to the genera *Bifidobacterium, Bacteroides*, and *Enterobacteriaceae* uncl. were often present at higher relative abundance in infant faeces than in milk, indicating the selection of these ASVs by the gut environment.

### Temporal changes in HMO concentrations and associations between milk HMOs and milk or faecal bacteria

**Figure 5.**
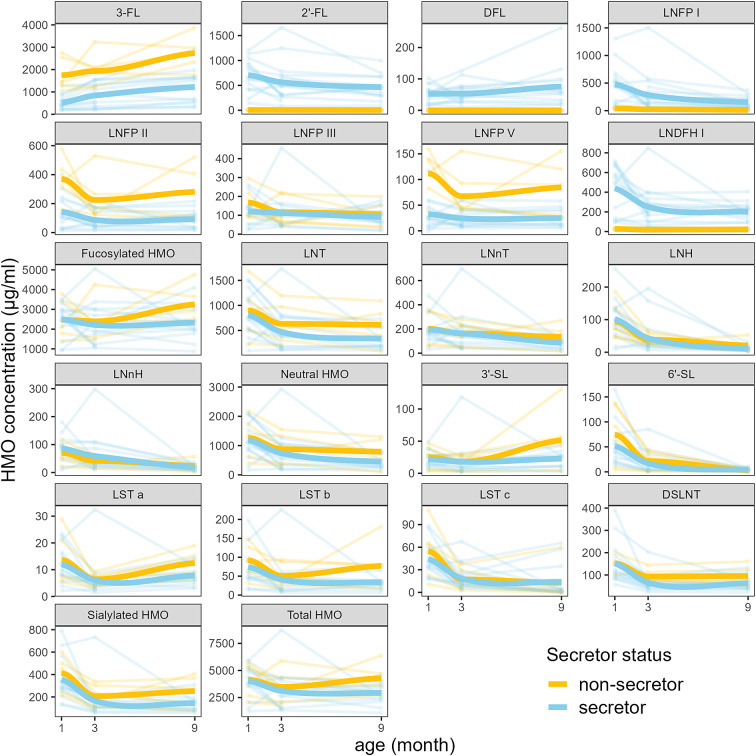
HMO concentration trajectories during the first nine months of lactation in milk of secretor and non-secretor mothers. The thick solid lines represent the trend lines plotted with a locally weighted scatterplot smoothing (LOESS).

Changes in the concentrations of HMOs during lactation were observed (Figure 5). Particularly, the concentration of 3-FL increased during the first nine months of lactation in the milk from secretor mothers (Supplementary Table 4). The concentrations of other HMOs showed either a decreasing trend or remained constant throughout the first nine months of lactation. On the other hand, we did not see significant differences in the HMO concentrations quantified in the milk from non-secretor mothers among samples collected at different time points (Supplementary Table 5). Besides the near absence of 2′-FL, milk from non-secretor mothers also contained a lower concentration of DFL, LNFP I, and LNDFH I, compared to milk from secretor mothers (Supplementary Table 6). Interestingly, there was no significant difference in the concentration of total fucosylated HMOs between milk samples grouped by maternal secretor status. This can be explained by the observed higher concentrations of 3-FL, LNFP II, and LNFP V in the milk of non-secretor mothers, compared to the milk from secretor mothers.

Despite the natural variation in HMO profiles, the secretor status and HMO concentrations had limited effect on the gut microbiota of breastfed infants at one, three, and nine months of age. Neither infant sex, birth place, milk collection method, maternal secretor status, nor individual HMOs significantly contributed to explaining the observed variation in microbiota composition of maternal milk and infant faeces after FDR correction (PERMANOVA, Supplementary Table 7). Moreover, we did not observe significant associations, after FDR correction for multiple comparisons, between the individual HMO concentrations and the bacterial ASVs in the maternal milk and infant faeces (Supplementary Table 8).

## Discussion

In this longitudinal study of a total of 23 mother–infant pairs, we observed that the microbiota in the maternal milk and infant gut developed in a temporal manner. The microbiota in the maternal milk samples was predominated by the genera *Streptococcus, Staphylococcus* and an unclassified genus within *Enterobacteriaceae*, in line with previous studies that reported human milk microbiota profiles (Urbaniak et al., 2016; Cabrera-Rubio et al., 2019; Fehr et al., 2020). Compared to milk at one and three months postpartum, a distinct composition of the milk microbiota was observed at nine months postpartum, characterized by an increased proportion of *Neisseria, Granulicatella, Haemophilus, Actinomyces, Veillonella, Gemella*, and *Streptococcus*, which are all bacterial genera that often colonize the oral cavity of infants (Dzidic et al., 2018).

Infancy represents an important stage in the development of the oral microbiota, marked by the eruption of teeth, exploratory mouthing behaviours, and the introduction of solid foods (Arishi et al., 2023). It should be noted that at nine months postpartum, the initiation of teeth eruption might provide an adhesion surface that favours the growth of common dental plaque bacteria in the mouth, such as *Granulicatella*, *Gemella, Actinomyces, Neisseria*, and *Streptococcus (*Aas et al., *2005).* The presence of infant oral bacteria in the maternal milk microbiota might be explained by the backward flow of breast milk into mammary ducts during infant suckling (Ramsay et al., 2004).

On the other hand, we observed that the microbiota in most of the infant faecal samples was either dominated by *Bifidobacterium* or showed a mixed proportion of bacterial genera, typical of this age in infancy (Borewicz et al., 2019). In the weaning period, when infants received complementary food, an increase in alpha-diversity was seen, and the changes in the faecal microbial composition were characterized by an increase in the relative abundance of members of the class *Clostridia*, including *Faecalibacterium, Blautia*, and *Anaerostipes*, similar to a previous study on infants at the same age group (Laursen et al., 2021). Although the composition of milk microbiota was distinct from that of the microbiota of infant faeces, some shared taxa at ASV level were identified. ASVs belonging to the genera *Bifidobacterium, Streptococcus, Staphylococcus, Veillonella*, and *Haemophilus* frequently co-occurred in the mother’s milk and the faeces from her own infant, in line with previous studies (Fehr et al., 2020; Laursen et al., 2021). It should be noted that the co-occurrence of bacterial ASVs in milk and infant faeces suggests mother-to-child microbial transmission, but is insufficient to confirm transmission during breastfeeding. Confirming this transmission during breastfeeding requires strain level resolution, which cannot be obtained from 16S rRNA gene amplicon sequence data.

Moreover, our data showed that the extent to which ASVs were shared within mother–infant pairs compared to unrelated pairs was only higher at one month postpartum, indicating that the colonization of the gut by ingested milk bacteria was more likely to occur at a younger age when the gut microbiota was less diverse. A previous study using a combined metagenomic-culture-based approach showed that strains of *Bifidobacterium* and *Staphylococcus* were frequently transmitted between maternal milk and infant stool at one month of age (Feehily et al., 2023). In addition, the same culturable bacterial strains of *Lactobacillus* were observed in breast milk and faeces of infants younger than three months of age (Martín et al., 2012).

Furthermore, the HMO composition changed over the course of lactation. Except for 3-FL, the concentration of other HMOs showed either no change or decreased over time, in line with previous studies (Borewicz et al., 2020; Lefebvre et al., 2020; Durham et al., 2021; Plows et al., 2021). It should be noted that while the milk of non-secretor mothers was lacking α-1,2-fucosylated HMOs (2′-FL, DFL, LNFP I, LNDFH I), a higher concentration of other fucosylated HMOs was seen, including 3-FL, LNFP II, and LNFP V (Durham et al., 2021; Menzel et al., 2021). Decorated fucose in α-1,2-fucosylated HMOs is removed by the α-1,2-fucosidase GH95, which is present in gut bacteria, including specific strains of *Bifidobacterium*, *Bacteroides*, and *Akkermansia (*Kiely et al., *2023).*

In line with other studies, we observed a limited effect of maternal secretor status on the composition of breast milk (Moossavi et al., 2019) or infant faecal microbiota (Borewicz et al., 2020; Laursen et al., 2021; Barnett et al., 2023). A previous study showed that the secretor status of the infant, but not maternal secretor status, was an important determinant of infant faecal microbiota (Thorman et al., 2023). Moreover, in concurrence with Laursen et al. (Laursen et al., 2021), our results showed a lack of significant association between HMO concentrations and infant faecal bacterial ASVs. In a larger study of 220 one-month-old infants, the concentrations of 6′-SL and LNH were associated with overall faecal microbiota composition, yet not with the proportion of specific gut bacteria (Barnett et al., 2023). This might be partially explained by the redundant and synergistic effects of HMOs, which could hinder the detection of associations between specific HMOs and their role in stimulating specific gut bacteria (Sprenger et al., 2022).

Our longitudinal study of a homogenous population of mother–infant pairs in the Netherlands provided an integrated overview of the temporal changes of HMOs and microbiota in breast milk and infant faeces, even though the small number of mother–infant dyads limited the statistical power of the analysis. Microbiota profiling was only performed on foremilk samples, since a previous study showed that the microbiota composition between fore- and hindmilk was similar (Laursen et al., 2021). However, we did not control for the variation in HMO concentrations of fore- and hindmilk. Despite its limitation in underestimating the abundance of skin bacteria (Meisel et al., 2016), the V4 universal primer pair was chosen for targeting both bacterial and archaeal 16S rRNA genes and to allow high-throughput analysis of faecal and milk microbiota. However, the microbiota analysis presented in this study focused only on bacterial composition due to low prevalence of archaea in human milk and no detection of this microbial group in infant faeces. The assessment of infant gut microbiota was approximated based on faecal material, and careful consideration should be given when interpreting these results, as faecal profiles may be a biased representation of the true colonic ecosystem diversity (Levitan et al., 2023). Furthermore, a larger sample size, a detailed measurement of dietary data, and the use of strain level analysis (e.g. combining shotgun metagenomics and cultivation-based approaches) are of importance in the design of future longitudinal studies investigating bacterial transmission via breastfeeding.

This study demonstrates that the concentration of milk oligosaccharides and the microbiota composition of milk and infant faeces changes between one and nine months postpartum. Shared bacteria in human milk and infant faeces within the mother–infant dyads suggest the importance of milk microbes in shaping the assembly of gut microbiota in an age-dependent fashion. Finally, considering the fact that we did not observe specific associations between bacterial taxa and HMO concentrations, it is tempting to speculate that different HMOs might exhibit overlapping roles in feeding the gut bacteria, regardless of the differences in HMO profiles determined by the maternal secretor status.

## Supporting information

Endika et al. supplementary material 1 Endika et al. supplementary material

Endika et al. supplementary material 2 Endika et al. supplementary material

Endika et al. supplementary material 3 Endika et al. supplementary material

## Data Availability

The data for this study have been deposited in the European Nucleotide Archive (ENA) at EMBL-EBI under accession number PRJEB64690.

## Abbreviations

ASV: Amplicon sequence variant
CAN: Acetonitrile
CLR: Centred log-ratio
ENA: European Nucleotide Archive
HMO: Human milk oligosaccharide
HPAEC-PAD: High-performance anion-exchange chromatography-pulsed amperometric detection
LOESS: Locally weighted scatterplot smoothing
PCA: Principal component analysis
PGC-LC-MS: Porous graphitized carbon-liquid chromatography mass spectrometry
SPE: Solid-phase extraction
STAR: Stool Transport and Recovery

## Supplementary material

The supplementary material for this article can be found at http://doi.org/10.1017/gmb.2024.5.
