# Supplementary material for: Seeding and feeding milestones: the role of human milk microbes and oligosaccharides in the temporal development of infant gut microbiota
Source: Gut Microbiome (Camb). 2024 May 31;5:e7. doi: 10.1017/gmb.2024.5 (PMC11706684; doi:10.1017/gmb.2024.5)
Supplement: Endika et al. supplementary material 1 — Endika et al. supplementary material [file S2632289724000057sup001.docx]

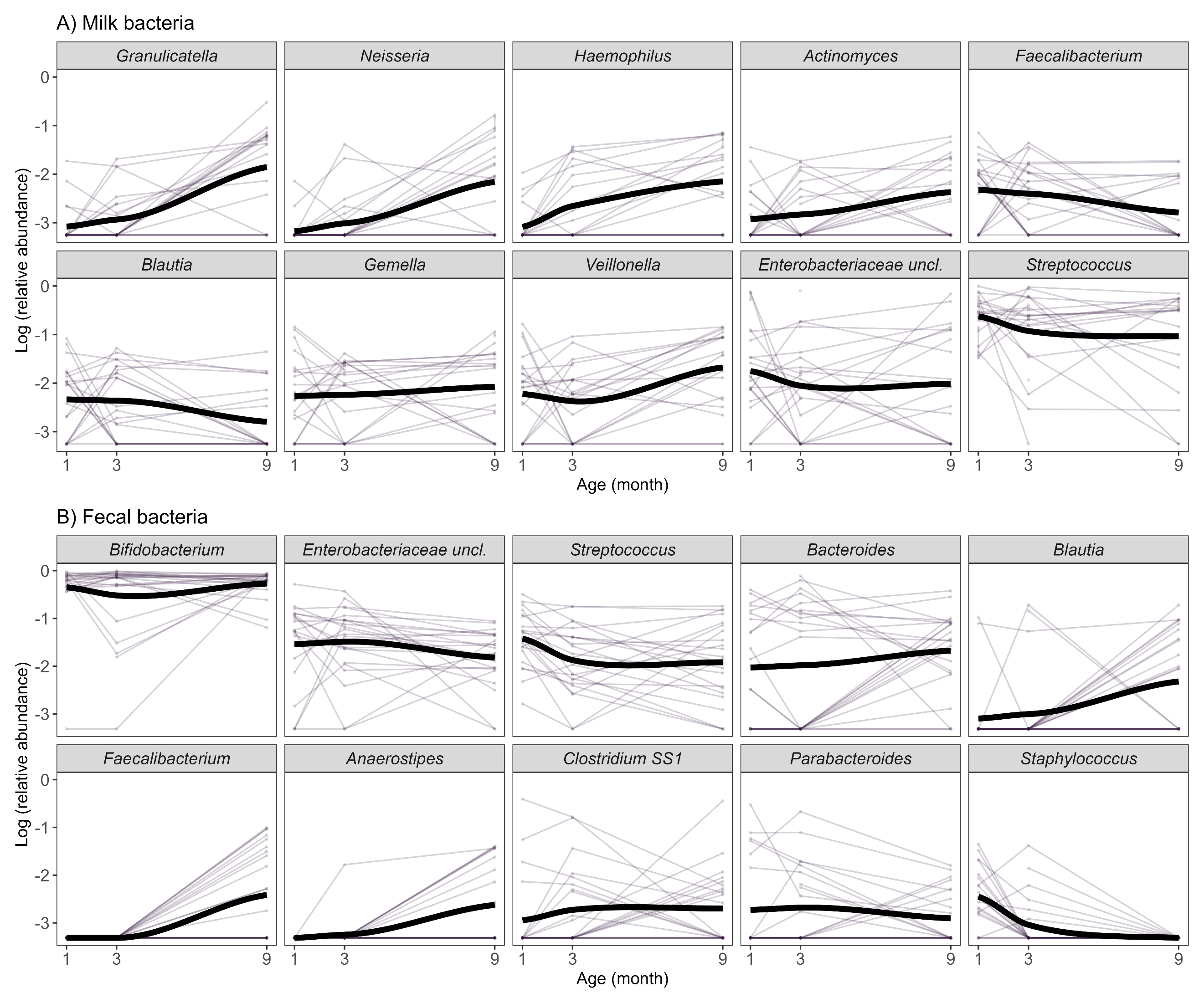
**Supplementary Material**

**Supplementary Figure 1.** The trajectories in the proportion of 10 genus-level taxa that contributed most to the observed variation in microbial composition in milk **(A)** and infant feces **(B)**. The solid lines represent the trend lines plotted with a locally-weighted scatterplot smoothing (LOESS).

**Supplementary Table S1.** List of Human Milk Oligosaccharides (HMOs) quantified in the current study

| **HMO Category** | **Name** | **Abbreviation** | **HMO Standards Supplier** |
| --- | --- | --- | --- |
| Fucosylated | 3-fucosyllactose | 3-FL | Dextra Laboratories (Reading, UK) |
|  | 2′-fucosyllactose | 2′-FL | Carbosynth Ltd (Compton, UK) |
|  | Difucosyllactose | DFL | ELICITYL (Crolles, France) |
|  | Lacto-N-fucopentaose I | LNFP I | Dextra Laboratories (Reading, UK) |
|  | Lacto-N-fucopentaose II | LNFP II | Dextra Laboratories (Reading, UK) |
|  | Lacto-N-fucopentaose III | LNFP III | Dextra Laboratories (Reading, UK) |
|  | Lacto-N-fucopentaose V | LNFP V | Glyko (San Francisco, CA, USA) |
|  | Lacto-N-difucohexaose I | LNDFH I | Carbosynth Ltd (Compton, UK) |
| Neutral | Lacto-N-tetraose | LNT | Carbosynth Ltd (Compton, UK) |
|  | Lacto-N-neotetraose | LNnT | Dextra Laboratories (Reading, UK) |
|  | Lacto-N-hexaose | LNH | Dextra Laboratories (Reading, UK) |
|  | Lacto-N-neohexaose | LNnH | Dextra Laboratories (Reading, UK) |
| Sialylated | 3′-sialyllactose | 3′-SL | Sigma-Aldrich (St. Louis, MO, USA) |
|  | 6′-sialyllactose | 6′-SL | Sigma-Aldrich (St. Louis, MO, USA) |
|  | Sialyl-lacto-N-tetraose a | LST a | Dextra Laboratories (Reading, UK) |
|  | Sialyl-lacto-N-tetraose b | LST b | Dextra Laboratories (Reading, UK) |
|  | Sialyl-lacto-N-tetraose c | LST c | Dextra Laboratories (Reading, UK) |
|  | Disialyllacto-N-tetraose | DSLNT | Carbosynth LTd (Compton, UK) |

**Supplementary Table S2.** Baby Carbs participant characteristics

|  |  | Total | Infant age group | | |
| --- | --- | --- | --- | --- | --- |
|  |  | (n = 23) | 1 month  (n = 21) | 3 months  (n = 23) | 9 months  (n = 23) |
|  |  |  |  |  |  |
| **Mother** |  |  |  |  |  |
| Age, y (SD) |  | 33.36 (2.92) |  |  |  |
| Pre-pregnancy BMI, n (%) | Normal | 20 (86.96) |  |  |  |
|  | Overweight | 3 (13.04) |  |  |  |
| Milk samples collected, n |  |  | 21 | 23 | 19 |
| Secretor status, n (%) | Secretor | 17 (73.91) | 15 (71.43) | 17 (73.91) | 14 (73.68) |
|  | Non-secretor | 6 (26.08) | 6 (28.57) | 6 (26.09) | 5 (26.32) |
| Collection method, n (%) | Milk pump |  | 16 (76.19) | 19 (82.61) | 17 (89.47) |
|  | Hand expression |  | 5 (23.81) | 4 (17.39) | 2 (10.53) |
| Mother health issues, n (%) | Cold symptoms |  | 2 (9.52) | 3 (13.04) | 4 (17.39) |
|  | Diarrhea |  | 1 (4.76) | 0 | 0 |
|  | Thrush |  | 0 | 1 (4.35) | 0 |
|  | None |  | 18 (85.71) | 19 (82.61) | 19 (82.61) |
| **Infant** |  |  |  |  |  |
| Sex, n (%) | Female | 12 (52.17) |  |  |  |
|  | Male | 11 (47.83) |  |  |  |
| Birth place, n (%) | Home | 11 (47.83) |  |  |  |
|  | Hospital | 10 (43.48) |  |  |  |
|  | Others | 2 (8.70) |  |  |  |
| Gestational age at birth (weeks), n (%) | 37 – 40 | 10 (43.48) |  |  |  |
|  | > 40 | 13 (56.52) |  |  |  |
| Birth weight in kg (SD) |  | 3.81 (0.51) |  |  |  |
| Age, d (SD) |  |  | 35.10 (6.10) | 97.09 (8.44) | 276.91 (5.62) |
| Fecal samples collected, n |  |  | 21 | 23 | 23 |
| Infant health issues, n (%) | Cold symptoms |  | 1 (4.76) | 8 (34.78) | 7 (30.43) |
|  | Diarrhea |  | 0 | 0 | 2 (8.70) |
|  | Thrush |  | 0 | 1 (4.35) | 0 |
|  | Constipation |  | 0 | 0 | 2 (8.70) |
|  | Colic |  | 1 (4.76) | 1 (4.35) | 0 |
|  | None |  | 19 (90.58) | 13 (56.52) | 12 (52.17) |

**Supplementary Table S3.** PERMANOVA test for significance of age group on microbiota composition

| Sample |  | Df | Sum Of Sqs | R2 | F | Pr(>F) |
| --- | --- | --- | --- | --- | --- | --- |
| Fecal microbiota | Age group | 2 | 414.8 | 0.11 | 3.97 | 1e-04 *** |
|  | Residual | 64 | 3339.1 | 0.89 |  |  |
|  | Total | 66 | 3753.9 | 1.00 |  |  |
| Milk microbiota | Age group | 2 | 518.2 | 0.09 | 3.13 | 1e-04 *** |
|  | Residual | 60 | 4968.9 | 0.91 |  |  |
|  | Total | 62 | 5487.1 | 1.00 |  |  |

| Human Milk Oligosaccharides (HMOs) | Average concentration (µg/ml ± SD) | | | | | | | | |
| --- | --- | --- | --- | --- | --- | --- | --- | --- | --- |
|  | Secretor | | |  | | |  | | |
|  | 1 month postpartum | 3 months postpartum | p | 1 month postpartum | 9 months postpartum | p | 3 months postpartum | 9 months postpartum | p |
| 3-FL | 499.54 ± 308.89 | 837.71 ± 482.67 | *** | 499.54 ± 308.89 | 1223.5 ± 750.44 | *** | 837.71 ± 482.67 | 1223.5 ± 750.44 | ** |
| 2′-FL | 713.81 ± 309.75 | 563.44 ± 393.09 | ns | 713.81 ± 309.75 | 464.47 ± 242.65 | * | 563.44 ± 393.09 | 464.47 ± 242.65 | * |
| DFL | 51.11 ± 26.64 | 53.21 ± 31.3 | ns | 51.11 ± 26.64 | 75.57 ± 62.9 | * | 53.21 ± 31.3 | 75.57 ± 62.9 | ns |
| LNFP I | 511.09 ± 366.53 | 283.71 ± 357.77 | * | 511.09 ± 366.53 | 152.82 ± 105.67 | * | 283.71 ± 357.77 | 152.82 ± 105.67 | * |
| LNFP II | 144.12 ± 110.82 | 87.5 ± 62.51 | ns | 144.12 ± 110.82 | 93.34 ± 62.55 | ns | 87.5 ± 62.51 | 93.34 ± 62.55 | ns |
| LNFP III | 118.65 ± 66.76 | 111.45 ± 97.13 | ns | 118.65 ± 66.76 | 90.71 ± 49.7 | ns | 111.45 ± 97.13 | 90.71 ± 49.7 | ns |
| LNFP V | 33.05 ± 29.03 | 24.4 ± 17.8 | ns | 33.05 ± 29.03 | 24.84 ± 16.17 | ns | 24.4 ± 17.8 | 24.84 ± 16.17 | ns |
| LNDFH I | 448.79 ± 231.01 | 251.15 ± 186.51 | * | 448.79 ± 231.01 | 201.55 ± 98.3 | ** | 251.15 ± 186.51 | 201.55 ± 98.3 | ns |
| Total Fucosylated HMOs | 852.53 ± 469.26 | 469.76 ± 408.25 | ns | 852.53 ± 469.26 | 337.15 ± 196.95 | ns | 469.76 ± 408.25 | 337.15 ± 196.95 | ns |
| LNT | 2520.16 ± 809.22 | 2212.57 ± 1016.85 | * | 2520.16 ± 809.22 | 2326.81 ± 844.52 | * | 2212.57 ± 1016.85 | 2326.81 ± 844.52 | * |
| LNnT | 195.79 ± 111.45 | 163.29 ± 159.03 | ns | 195.79 ± 111.45 | 84.94 ± 40.12 | ** | 163.29 ± 159.03 | 84.94 ± 40.12 | *** |
| LNH | 111.49 ± 71.07 | 43.3 ± 52.78 | ** | 111.49 ± 71.07 | 11.03 ± 6.84 | *** | 43.3 ± 52.78 | 11.03 ± 6.84 | *** |
| LNnH | 86.91 ± 43.1 | 58.82 ± 68.78 | ns | 86.91 ± 43.1 | 17.77 ± 9.56 | *** | 58.82 ± 68.78 | 17.77 ± 9.56 | *** |
| Total Neutral (core) HMOs | 1246.72 ± 636.04 | 735.16 ± 665.65 | * | 1246.72 ± 636.04 | 450.89 ± 223.17 | ** | 735.16 ± 665.65 | 450.89 ± 223.17 | ** |
| 3′-SL | 21.29 ± 12.2 | 17.95 ± 27.24 | ns | 21.29 ± 12.2 | 22.74 ± 13.68 | ns | 17.95 ± 27.24 | 22.74 ± 13.68 | ns |
| 6′-SL | 51.95 ± 39.16 | 16.86 ± 21.49 | *** | 51.95 ± 39.16 | 4.28 ± 2.96 | *** | 16.86 ± 21.49 | 4.28 ± 2.96 | *** |
| LST a | 11.74 ± 6.48 | 6.02 ± 7.03 | * | 11.74 ± 6.48 | 7.81 ± 3.53 | * | 6.02 ± 7.03 | 7.81 ± 3.53 | * |
| LST b | 75.45 ± 46.78 | 40.96 ± 50.66 | * | 75.45 ± 46.78 | 33.73 ± 18.47 | ** | 40.96 ± 50.66 | 33.73 ± 18.47 | ns |
| LST c | 45.37 ± 21.35 | 18.56 ± 17.04 | ** | 45.37 ± 21.35 | 13.91 ± 22.25 | * | 18.56 ± 17.04 | 13.91 ± 22.25 | ns |
| DSLNT | 148.67 ± 90.13 | 65.7 ± 39.97 | *** | 148.67 ± 90.13 | 62.05 ± 30.72 | *** | 65.7 ± 39.97 | 62.05 ± 30.72 | ns |
| Total Sialylated HMOs | 354.48 ± 179.67 | 166.06 ± 155.91 | *** | 354.48 ± 179.67 | 144.52 ± 70.77 | *** | 166.06 ± 155.91 | 144.52 ± 70.77 | ns |
| Total HMOs | 4121.37 ± 1462.63 | 3113.79 ± 1772.04 | ns | 4121.37 ± 1462.63 | 2922.22 ± 974.35 | ns | 3113.79 ± 1772.04 | 2922.22 ± 974.35 | ns |

**Supplementary Table S4.** Average HMO concentrations in breast milk of secretor mothers and differences in concentrations over time

Comparison of the means of HMO concentrations between different age group were evaluated by paired Wilcoxon signed-rank tests. Significant differences are indicated by *p < 0.05. **p < 0.01, ***p < 0.001, **** p < 0.0001. ns = non-significant.

**Supplementary Table S5.** Average HMO concentrations in breast milk of non-secretor mothers and differences in concentrations over time

| Human Milk Oligosaccharides (HMOs) | Average concentration (µg/ml ± SD) | | | | | | | | |
| --- | --- | --- | --- | --- | --- | --- | --- | --- | --- |
|  | Non-secretor | | |  | | |  | | |
|  | 1 month postpartum | 3 months postpartum | p | 1 month postpartum | 9 months postpartum | p | 3 months postpartum | 9 months postpartum | p |
| 3-FL | 1751.45 ± 716.6 | 1937.42 ± 749.01 | ns | 1751.45 ± 716.6 | 2738.65 ± 696.99 | ns | 1937.42 ± 749.01 | 2738.65 ± 696.99 | ns |
| 2′-FL | 6.95 ± 5.57 | 7.03 ± 6.45 | ns | 6.95 ± 5.57 | 7.27 ± 7.46 | ns | 7.03 ± 6.45 | 7.27 ± 7.46 | ns |
| DFL | 0 ± 0 | 0 ± 0 | NA | 0 ± 0 | 0 ± 0 | NA | 0 ± 0 | 0 ± 0 | NA |
| LNFP I | 40.81 ± 21.3 | 23.6 ± 16.49 | ns | 40.81 ± 21.3 | 12.89 ± 5.88 | ns | 23.6 ± 16.49 | 12.89 ± 5.88 | ns |
| LNFP II | 370.21 ± 139.46 | 225.14 ± 158.88 | ns | 370.21 ± 139.46 | 250.12 ± 181.87 | ns | 225.14 ± 158.88 | 250.12 ± 181.87 | ns |
| LNFP III | 166.75 ± 82.08 | 116.46 ± 79.28 | ns | 166.75 ± 82.08 | 94.48 ± 69.9 | ns | 116.46 ± 79.28 | 94.48 ± 69.9 | ns |
| LNFP V | 111.83 ± 45.57 | 67.38 ± 48.68 | ns | 111.83 ± 45.57 | 75.75 ± 54.56 | ns | 67.38 ± 48.68 | 75.75 ± 54.56 | ns |
| LNDFH I | 26.88 ± 9.03 | 19.25 ± 12.3 | ns | 26.88 ± 9.03 | 18.98 ± 13.42 | ns | 19.25 ± 12.3 | 18.98 ± 13.42 | ns |
| Total Fucosylated HMOs | 898.62 ± 500.52 | 632.93 ± 383.62 | ns | 898.62 ± 500.52 | 577.73 ± 358.73 | ns | 632.93 ± 383.62 | 577.73 ± 358.73 | ns |
| LNT | 2474.88 ± 935.96 | 2396.28 ± 1004.84 | ns | 2474.88 ± 935.96 | 3198.15 ± 881.82 | ns | 2396.28 ± 1004.84 | 3198.15 ± 881.82 | ns |
| LNnT | 201.08 ± 127.33 | 163.12 ± 96.45 | ns | 201.08 ± 127.33 | 131.09 ± 86.38 | ns | 163.12 ± 96.45 | 131.09 ± 86.38 | ns |
| LNH | 94.2 ± 56.41 | 38.89 ± 17.74 | ns | 94.2 ± 56.41 | 20.94 ± 17.15 | ns | 38.89 ± 17.74 | 20.94 ± 17.15 | ns |
| LNnH | 70.21 ± 34.58 | 41.53 ± 19.74 | ns | 70.21 ± 34.58 | 23.86 ± 18.15 | ns | 41.53 ± 19.74 | 23.86 ± 18.15 | ns |
| Total Neutral (core) HMOs | 1264.11 ± 664.72 | 876.47 ± 496.26 | ns | 1264.11 ± 664.72 | 753.62 ± 450.67 | ns | 876.47 ± 496.26 | 753.62 ± 450.67 | ns |
| 3′-SL | 25.07 ± 17.23 | 17.9 ± 12.3 | ns | 25.07 ± 17.23 | 46.26 ± 45.42 | ns | 17.9 ± 12.3 | 46.26 ± 45.42 | ns |
| 6′-SL | 74.07 ± 59.02 | 21.48 ± 18.25 | ns | 74.07 ± 59.02 | 3.7 ± 2.9 | ns | 21.48 ± 18.25 | 3.7 ± 2.9 | ns |
| LST a | 13.6 ± 9.49 | 6.4 ± 2.37 | ns | 13.6 ± 9.49 | 11.86 ± 4.81 | ns | 6.4 ± 2.37 | 11.86 ± 4.81 | ns |
| LST b | 91.43 ± 42.91 | 51.01 ± 31.99 | ns | 91.43 ± 42.91 | 69.52 ± 60.81 | ns | 51.01 ± 31.99 | 69.52 ± 60.81 | ns |
| LST c | 54.46 ± 35.12 | 17.27 ± 14.17 | ns | 54.46 ± 35.12 | 10.94 ± 24.02 | ns | 17.27 ± 14.17 | 10.94 ± 24.02 | ns |
| DSLNT | 152.26 ± 31.71 | 93.34 ± 29.64 | ns | 152.26 ± 31.71 | 90.69 ± 42.44 | ns | 93.34 ± 29.64 | 90.69 ± 42.44 | ns |
| Total Sialylated HMOs | 410.90 ± 148.97 | 207.40 ± 99.25 | ns | 410.90 ± 148.97 | 232.97 ± 136.84 | ns | 207.40 ± 99.25 | 232.97 ± 136.84 | ns |
| Total HMOs | 4149.89 ± 1537.28 | 3480.15 ± 1499.36 | ns | 4149.89 ± 1537.28 | 4184.74 ± 1254.85 | ns | 3480.15 ± 1499.36 | 4184.74 ± 1254.85 | ns |

Comparison of the means of HMO concentrations between different age group were evaluated by paired Wilcoxon signed-rank tests. Significant differences are indicated by *p < 0.05. **p < 0.01, ***p < 0.001, **** p < 0.0001. ns = non-significant. NA = not available.

**Supplementary Table S6.** Average concentration of individual and grouped HMOs, grouped by maternal secretor status per age group

| Human Milk Oligosaccharides (HMOs) | Average concentration (µg/ml ± SD) | | | | | | | | |
| --- | --- | --- | --- | --- | --- | --- | --- | --- | --- |
|  | 1 month postpartum | | | 3 months postpartum | | | 9 months postpartum | | |
|  | Secretor | Non-secretor | p | Secretor | Non-secretor | p | Secretor | Non-secretor | p |
| 3-FL | 499.54 ± 308.89 | 1751.45 ± 716.6 | *** | 837.71 ± 482.67 | 1937.42 ± 749.01 | ** | 1223.5 ± 750.44 | 2738.65 ± 696.99 | ** |
| 2′-FL | 713.81 ± 309.75 | 6.95 ± 5.57 | **** | 563.44 ± 393.09 | 7.03 ± 6.45 | **** | 464.47 ± 242.65 | 7.27 ± 7.46 | **** |
| DFL | 51.11 ± 26.64 | 0 ± 0 | **** | 53.21 ± 31.3 | 0 ± 0 | **** | 75.57 ± 62.9 | 0 ± 0 | ** |
| LNFP I | 511.09 ± 366.53 | 40.81 ± 21.3 | *** | 283.71 ± 357.77 | 23.6 ± 16.49 | *** | 152.82 ± 105.67 | 12.89 ± 5.88 | *** |
| LNFP II | 144.12 ± 110.82 | 370.21 ± 139.46 | ** | 87.5 ± 62.51 | 225.14 ± 158.88 | * | 93.34 ± 62.55 | 250.12 ± 181.87 | * |
| LNFP III | 118.65 ± 66.76 | 166.75 ± 82.08 | ns | 111.45 ± 97.13 | 116.46 ± 79.28 | ns | 90.71 ± 49.7 | 94.48 ± 69.9 | ns |
| LNFP V | 33.05 ± 29.03 | 111.83 ± 45.57 | ** | 24.4 ± 17.8 | 67.38 ± 48.68 | ** | 24.84 ± 16.17 | 75.75 ± 54.56 | * |
| LNDFH I | 448.79 ± 231.01 | 26.88 ± 9.03 | **** | 251.15 ± 186.51 | 19.25 ± 12.3 | **** | 201.55 ± 98.3 | 18.98 ± 13.42 | **** |
| Total Fucosylated HMOs | 852.53 ± 469.26 | 898.62 ± 500.52 | ns | 469.76 ± 408.25 | 632.93 ± 383.62 | ns | 337.15 ± 196.95 | 577.73 ± 358.73 | ns |
| LNT | 2520.16 ± 809.22 | 2474.88 ± 935.96 | ns | 2212.57 ± 1016.85 | 2396.28 ± 1004.84 | ns | 2326.81 ± 844.52 | 3198.15 ± 881.82 | ns |
| LNnT | 195.79 ± 111.45 | 201.08 ± 127.33 | ns | 163.29 ± 159.03 | 163.12 ± 96.45 | ns | 84.94 ± 40.12 | 131.09 ± 86.38 | ns |
| LNH | 111.49 ± 71.07 | 94.2 ± 56.41 | ns | 43.3 ± 52.78 | 38.89 ± 17.74 | ns | 11.03 ± 6.84 | 20.94 ± 17.15 | ns |
| LNnH | 86.91 ± 43.1 | 70.21 ± 34.58 | ns | 58.82 ± 68.78 | 41.53 ± 19.74 | ns | 17.77 ± 9.56 | 23.86 ± 18.15 | ns |
| Total Neutral (core) HMOs | 1246.72 ± 636.04 | 1264.11 ± 664.72 | ns | 735.16 ± 665.65 | 876.47 ± 496.26 | ns | 450.89 ± 223.17 | 753.62 ± 450.67 | ns |
| 3′-SL | 21.29 ± 12.2 | 25.07 ± 17.23 | ns | 17.95 ± 27.24 | 17.9 ± 12.3 | ns | 22.74 ± 13.68 | 46.26 ± 45.42 | ns |
| 6′-SL | 51.95 ± 39.16 | 74.07 ± 59.02 | ns | 16.86 ± 21.49 | 21.48 ± 18.25 | ns | 4.28 ± 2.96 | 3.7 ± 2.9 | ns |
| LST a | 11.74 ± 6.48 | 13.6 ± 9.49 | ns | 6.02 ± 7.03 | 6.4 ± 2.37 | ns | 7.81 ± 3.53 | 11.86 ± 4.81 | ns |
| LST b | 75.45 ± 46.78 | 91.43 ± 42.91 | ns | 40.96 ± 50.66 | 51.01 ± 31.99 | ns | 33.73 ± 18.47 | 69.52 ± 60.81 | ns |
| LST c | 45.37 ± 21.35 | 54.46 ± 35.12 | ns | 18.56 ± 17.04 | 17.27 ± 14.17 | ns | 13.91 ± 22.25 | 10.94 ± 24.02 | ns |
| DSLNT | 148.67 ± 90.13 | 152.26 ± 31.71 | ns | 65.7 ± 39.97 | 93.34 ± 29.64 | ns | 62.05 ± 30.72 | 90.69 ± 42.44 | ns |
| Total Sialylated HMOs | 354.48 ± 179.67 | 410.90 ± 148.97 | ns | 166.06 ± 155.91 | 207.40 ± 99.25 | ns | 144.52 ± 70.77 | 232.97 ± 136.84 | ns |
| Total HMOs | 4121.37 ± 1462.63 | 4149.89 ± 1537.28 | ns | 3113.79 ± 1772.04 | 3480.15 ± 1499.36 | ns | 2922.22 ± 974.35 | 4184.74 ± 1254.85 | ns |

Comparison of the means of HMO concentrations between secretor and non-secretor group were evaluated by Mann–Whitney or t-tests (depending on normality of data distribution). Significant differences are indicated by *p < 0.05. **p < 0.01, ***p < 0.001, **** p < 0.0001. ns = non-significant.
